# Association of common variation in *ADD3* and *GPC1* with biliary atresia susceptibility

**DOI:** 10.18632/aging.103067

**Published:** 2020-04-21

**Authors:** Mei-Rong Bai, Wei-Bo Niu, Ying Zhou, Yi-Ming Gong, Yan-Jiao Lu, Xian-Xian Yu, Zhi-Liang Wei, Wenjie Wu, Huan-Lei Song, Wen-Wen Yu, Bei-Lin Gu, Wei Cai, Xun Chu

**Affiliations:** 1 Department of Pediatric Surgery, Xinhua Hospital, School of Medicine, Shanghai Jiao Tong University, Shanghai, China; 2 Shanghai Key Laboratory of Pediatric Gastroenterology and Nutrition, Shanghai, China; 3 Shanghai Institute of Pediatric Research, Shanghai, China

**Keywords:** biliary atresia, *ADD3*, *GPC1*, single nucleotide polymorphism, association

## Abstract

Biliary atresia (BA) is an idiopathic neonatal cholestatic disease. Recent genome-wide association study (GWAS) revealed that common variation of *ADD3*, *GPC1*, *ARF6*, and *EFEMP1* gene was associated with BA susceptibility. We aimed to evaluate the association of these genes with BA in Chinese population. Twenty single nucleotide polymorphisms (SNPs) in these four genes were genotyped in 340 BA patients and 1,665 controls. Three SNPs in *ADD3* were significantly associated with BA, and rs17095355 was the top SNP (*P*_Allele_ = 3.23×10^-6^). Meta-analysis of published data and current data indicated that rs17095355 was associated with BA susceptibility in Asians and Caucasians. Three associated SNPs were expression quantitative trait loci (eQTL) for *ADD3*. Two *GPC1* SNPs in high linkage disequilibrium (LD) showed nominal association with BA susceptibility (*P*_Allele_ = 0.03 for rs6707262 and *P*_Allele_ = 0.04 for rs6750380), and were eQTL of *GPC1*. Haplotype harboring these two SNPs almost reached the study-wide significance (*P* = 0.0035). No association for *ARF6* and *EFEMP1* was found with BA risk in the current population. Our study validated associations of *ADD3* and *GPC1* SNPs with BA risk in Chinese population and provided evidence of epistatic contributions of genetic factors to BA susceptibility.

## INTRODUCTION

Biliary atresia (BA) is a devastating inflammatory and fibro-obliterative disease of the infant biliary tree involving extra- and intrahepatic bile ducts which invariably leads, if left untreated, to cholestasis and hepatic fibrosis even progresses to liver cirrhosis and eventually liver failure [1]. The most effective treatment of choice is palliative surgery (Kasai operation) and the majority of patients would still need liver transplantation later in life due to the progressive intrahepatic bile ducts injury [2]. The majority of BA (about 80 % of cases) occurs as an isolated defect without any associated disorders, and 10%-20% of patients with at least one major congenital malformation [3, 4]. The occurrence of BA has geographical, seasonal and gender differences. The incidence rate of BA in western countries is about (0.5 to 0.8)/10,000, which is lower than Asians. The incidence is 1.5/10,000 in Taiwan, and about 1.1/10,000 in Japanese population [5, 6]. BA exhibits a slight gender bias, with a female to male ratio about 1.25:1 [7]. It is likely to be a multifactorial disease, in that environmental and genetic interaction underlies its pathogenesis. The genetic basis of BA is quite complicated. It was found that the disease could be inherited in a dominant or recessive pattern but more probably was a polygenic condition with incomplete penetrance, genetic heterogeneity and variable clinical manifestations [3, 8]. In the past twenty years, a number of risk genes were found [9–16]. Recent genome-wide association studies (GWASs) revealed that variants in adducing-3 (*ADD3*), glypican-1 (*GPC1*), adenosine diphosphate-ribosylation factor-6 (*ARF6*) and epidermal growth factor-containing fibulin-like extracellular matrix protein 1 (*EFEMP1*) were associated with BA susceptibility [9, 10, 12, 16].

A previous GWAS in Chinese population firstly identified a susceptibility locus for BA on 10q24.2 with rs17095355 as the lead single nucleotide polymorphism (SNP), which is located in the intergenic region between the X-prolyl aminopeptidase 1 (*XPNPEP1*) and *ADD3* genes [9]. The association was then validated in Thai, Chinese and European population [11, 17–21]. Further study in model organism revealed that both *xpnpep1* and *add3a* were expressed in the liver of developing zebrafish, only knockdown of *add3a* produced intrahepatic defects and decreased biliary function by activating Hedgehog signaling [22]. Chromosome 2q37 was identified as a potential susceptibility region for BA in a GWAS and continued fine-mapping indicated *GPC1* as a susceptibility gene [10, 23]. Disruption of *gpc1* in zebrafish led to biliary defects for overactivation of Hedgehog signaling [23]. Two common SNPs in *GPC1* were subsequently investigated in a Chinese case-control sample-set containing 134 cases and 618 controls, which found a significant association with rs2292832 and a marginal effect with rs3828336 [24]. A GWAS with 80 Caucasian BA cases and 2,818 controls found SNPs rs3126184 and rs10140366 in the 3′ flanking region of *ARF6* were associated with BA risk [12]. Knockdown of the two zebrafish homologs, *arf6a* and *arf6b*, caused a sparse intrahepatic biliary network, several biliary epithelial cell defects, and poor bile excretion to the gall bladder [12]. *EFEMP1* was found association with BA in a recent European-American population-based GWAS including 343 isolated BA patients and 1,716 controls, which was validated an independent European-American cohort including 156 patients with BA and 212 genetically-matched controls [16]. RNA expression analysis and immunohistochemistry analysis demonstrated that expression of *EFEMP1* was higher in BA patients than in controls [16].

With the aim to comprehensively investigate these newly identified susceptibility genes from recent GWASs, we conducted a case-control study in Chinese population consisting of 340 patients and 1,665 controls. Since *ADD3* variants were repeatedly studied, we performed a meta-analysis for BA association with the top SNP rs17095355. We also explored the functional consequences of associated SNPs via bioinformatics methods.

## RESULTS

### Case-control association study

Detailed clinical information and biochemical indexes of 340 BA patients are shown in Table 1. A total of 340 cases and 1,665 controls were genotyped for 20 SNPs. Two SNPs (rs10140366 and rs2292832) were filtered out for failure in assays. Seven samples were excluded for further analysis for genotyping missing rates ≥ 5%. The genotypes of the remaining 18 SNPs were conformed to Hardy-Weinsberg equilibrium (HWE) (*P* > 0.05) and the minor allele frequencies (MAFs) were all above 0.01. The allele and genotype frequencies are shown in Table 2 and Table 3.

**Table 1:** Clinical information and biochemical indexes of BA patients.

**Variables**	**BA patients**
Male/Female	192 / 125
Age (month)	2.20 ± 0.09
bile acid (μmol/L)	128.62 ± 2.98
ALT (IU/L)	168.72 ± 6.29
AST (IU/L)	257.66 ± 8.31
ALP (IU/L)	567.75 ± 12.82
GGT (IU/L)	581.64 ± 27.79
TB (μmol/L)	166.01 ± 3.32
DB (μmol/L)	115.70 ± 2.40

Data are means ± SEM; SEM: standard error of the mean; BA: Biliary atresia; ALT: Alanine aminotransferase; AST: Aspartate aminotransferase; ALP: Alkaline phosphatase; GGT: Gamma-glutamyl transpeptidase; TB: Total bilirubin; DB: Direct bilirubin.

**Table 2:** Case-control association tests for SNPs of *ADD3*, *GPC1*, *ARF6* and *EFEMP* in 333 BA patients and 1,665 controls.

**CHR**	**BP**	**SNP**	**Gene**	**Functional annotation**	**A1/A2**	**Minor Allele Frequency**		**Allelic *P* value**	**OR (95% CI)**
						**Cases**	**Controls**		
2	56108333	rs1346786	*EFEMP1*	intron	G/A	0.121	0.141	0.164	0.84(0.65- 1.08)
2	56115834	rs11125609	*EFEMP1*	intron	A/G	0.440	0.457	0.426	0.93(0.79- 1.11)
2	56118046	rs10865291	*EFEMP1*	intron	G/A	0.200	0.222	0.211	0.88(0.71- 1.08)
2	56120853	rs1430193	*EFEMP1*	intron	A/T	0.087	0.090	0.846	0.97(0.72- 1.30)
2	241359706	rs1316479	*GPC1*	5'upstream	A/G	0.076	0.090	0.232	0.83(0.60- 1.13)
2	241362669	rs6750380	*GPC1*	5'upstream	G/A	0.434	0.392	0.041	1.19(1.01- 1.41)
2	241371065	rs6707262	*GPC1*	5'upstream	G/A	0.438	0.392	0.027	1.21(1.02- 1.43)
2	241382083	rs7577243	*GPC1*	intron	G/A	0.429	0.393	0.083	1.16(0.98- 1.37)
2	241385681	rs11692341	*GPC1*	intron	G/A	0.476	0.445	0.139	1.13(0.96- 1.34)
2	241392025	rs13431676	*GPC1*	intron	A/G	0.017	0.018	0.745	0.90(0.47- 1.72)
2	241403957	rs12695020	*GPC1*	intron	A/G	0.317	0.325	0.687	0.96(0.81- 1.15)
2	241404499	rs2228327	*GPC1*	synonymous	A/G	0.129	0.124	0.700	1.05(0.82- 1.35)
2	241405528	rs2228331	*GPC1*	missense	G/A	0.326	0.336	0.610	0.95(0.80- 1.14)
2	241419842	rs6739196	*GPC1*	intron	A/G	0.048	0.051	0.747	0.94(0.64- 1.38)
10	111735750	rs17095355	*ADD3*	intron	T/C	0.494	0.397	3.23×10^-6^	1.49(1.26- 1.76)
10	111757674	rs10509906	*ADD3*	intron	C/G	0.173	0.235	4.78×10^-4^	0.68(0.55- 0.85)
10	111846687	rs2501577	*ADD3*	intron	G/A	0.464	0.389	2.91×10^-4^	1.36(1.15- 1.61)
14	50322886	rs3126184	*ARF6*	5'upstream	G/A	0.030	0.037	0.401	0.81(0.50- 1.32)

CHR: Chromosome; BP: Base pair; SNP: Single Nucleotide Polymorphism; OR: odds ratio; CI: confidence interval.

**Table 3:** Genotype distributions of *ADD3* associated SNPs (rs17095355, rs10509906 and rs2501577) and *GPC1* SNPs (rs6750380 and rs6707262) in BA patients and healthy controls.

**SNP**	**Genotype**	**Genotype distribution N (%)**			***P* value**		
		**Case**	**Control**		**Genotype**	**Dominant**	**Recessive**
rs17095355	TT	76(22.8)	275(16.5)		1.15×10^-5^	4.34×10^-6^	5.77×10^-3^
	TC	177(53.2)	771(46.3)				
	CC	80(24.0)	619(37.2)				
rs10509906	CC	11(3.3)	98(5.9)		2.46×10^-3^	8.57×10^-4^	0.058
	CG	93(27.9)	584(35.1)				
	GG	229(68.8)	981(59.0)				
rs2501577	GG	66(19.8)	262(15.7)		5.88×10^-4^	1.39×10^-4^	0.066
	GA	177(53.2)	770(46.2)				
	AA	90(27.0)	633(38.0)				
rs6750380	GG	69(20.7)	263(15.8)		0.078	0.218	0.028
	GA	151(45.3)	777(46.7)				
	AA	113(33.9)	624(37.5)				
rs6707262	GG	71(21.2)	263(15.8)		0.043	0.203	0.014
	GA	150(45.0)	780(46.9)				
	AA	112(33.6)	621(37.3)				

SNP: Single Nucleotide Polymorphism.

**Figure 1:**
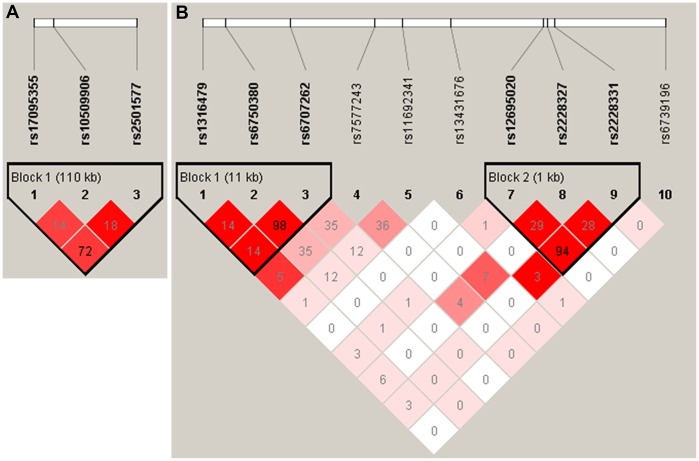
The linkage disequilibrium (LD) patterns of SNPs in *ADD3* (**A**) and *GPC1* (**B**). Haplotype blocks in *ADD3* and *GPC1* were defined according to the default method of Haploview. The numbers in the boxes are the pairwise correlation coefficient r^2^ between respective SNPs. *r^2^* values of 1 represent complete LD, *r^2^* values greater than 0.8 represent strong evidence of LD, *r^2^* values of 0.2 – 0.8 represent inconclusive LD, and *r^2^* less than 0.2 represent negligible evidence of LD The *r^2^* value between rs6750380 and rs6707262 of was 0.98.

All three tag SNPs of *ADD3* showed significant association (Table 2), rs17095355 (odds ratio (OR) = 1.49, 95% confidence interval (95% CI) = 1.26-1.76; *P*_Allele_ = 3.23×10^-6^), rs10509906 (OR = 0.68, 95% CI = 0.55-0.85; *P*_Allele_ = 4.78×10^-4^) and rs2501577 (OR = 1.36, 95% CI = 1.15-1.61; *P*_Allele_ = 2.91×10^-4^). The genotype frequency of these three SNPs in BA patients were also significantly different from those in controls (*P*_Genotypic- rs17095355_ = 1.15×10^-5^; *P*_Genotypic- rs10509906_ = 2.46×10^-3^; *P*_Genotypic- rs2501577_ = 5.88×10^-4^; Table 3). Analysis of model of inheritance for three SNPs showed a dominant model had the most significant effect on BA in the current population (rs17095355, *P*_Dominant_ = 4.34×10^-6^; rs10509906, *P*_Dominant_ = 8.57×10^-4^; rs2501577, *P*_Dominant_ = 1.39×10^-4^; Table 3). Linkage disequilibrium (LD) analysis showed the top SNP rs17095355 were in moderate LD with rs2501577 (r^2^ = 0.72), while in low LD with rs10509906 (r^2^ = 0.14) (Figure 1A). Conditional logistic analysis found no SNPs were significantly associated with disease risk after adjusting for rs17095355 effect (*P* > 0.05), suggesting that rs17095355 could solely account for *ADD3* association signal.

We further investigated whether *ADD3* SNP haplotypes were associated with BA susceptibility. Three associated SNPs of *ADD3* constructed a haplotype block. The frequency of haplotype rs17095355T - rs10509906G - rs2501577G in cases was significantly higher than that in controls (44% vs 36%, *P* = 4.86×10^-5^, OR = 1.42, 95% CI = 1.20-1.68; Table 4). Haplotype rs17095355C - rs10509906C - rs2501577A showed significant protective effect with *P* = 1.00×10^-4^ (16% in cases vs 22% in controls; OR = 0.65, 95% CI = 0.52- 0.81; Table 4).

**Table 4:** Association of ADD3 haplotypes constructed by rs17095355, rs10509906 and rs2501577.

**Haplotypes**	**Frequency**		**OR(95%CI)**	***P* value**
	**Cases**	**Controls**		
TGG	0.44	0.36	1.42(1.20-1.68)	4.86×10^-5^
CGA	0.33	0.35	0.90(0.76-1.08)	0.25
CCA	0.16	0.22	0.65(0.52-0.81)	1.00×10^-4^
TGA	0.04	0.03	1.30(0.82-2.05)	0.25
CGG	0.02	0.03	0.80(0.47-1.36)	0.38
TCA	0.02	0.01	1.51(0.79-2.89)	0.23

OR: odds ratio; CI: confidence interval.

Two SNPs in *GPC1* showed nominal association with BA susceptibility, rs6707262 (OR = 1.21, 95% CI = 1.02-1.43; *P*_Allele_ = 0.03; Table 2) and rs6750380 (OR = 1.19, 95% CI = 1.01-1.41, *P*_Allele_ = 0.04; Table 2). However, the two SNPs could not reach study-wide significance (0.05/18 = 0.0027). The genotype distribution of rs6707262 was nominally different between cases and controls (*P*_Genotypic_ = 0.043; Table 3). Haplotype analysis revealed these two SNPs and an adjacent SNP (rs1316479) constructed a haplotype block, and haplotype rs1316479G - rs6750380G - rs6707262G almost reached the study-wide significance (*P* = 0.0035) (Table 5). These two SNPs were in nearly perfect LD (r^2^ = 0.98), suggesting that they represent a same signal (Figure 1B). These data indicated common genetic variation of *GPC1* contributed to BA susceptibility in Chinese population. The previously associated *ARF6* SNP rs3126184 showed no significance in our samples (Table 2). The frequencies of rs3126184 allele T were 0.030 in cases and 0.037 in controls in current Chinese population. However, it was more frequent with 0.29 in cases and 0.13 in controls in Caucasian [12]. We found no associations of four previously reported risk SNPs of *EFEMP1* with BA susceptibility in current samples. The allele frequencies in healthy controls of these four SNPs were different between current study and the European-American cohort, where the associations were firstly discovered [16]. But the effect direction of three SNPs was consistent with that in previous study (Supplementary Table 1).

**Table 5:** Association of GPC1 haplotypes constructed by rs1316479, rs6750380 and rs6707262.

**Haplotypes**	**Frequency**		**OR(95%CI)**	***P* value**
	**Cases**	**Controls**		
GAA	0.562	0.606	0.83(0.70-0.98)	0.0327
GGG	0.356	0.299	1.30(1.09-1.54)	0.0035
AGG	0.078	0.090	0.85(0.62-1.16)	0.2874

OR: odds ratio; CI: confidence interval.

**Figure 2:**
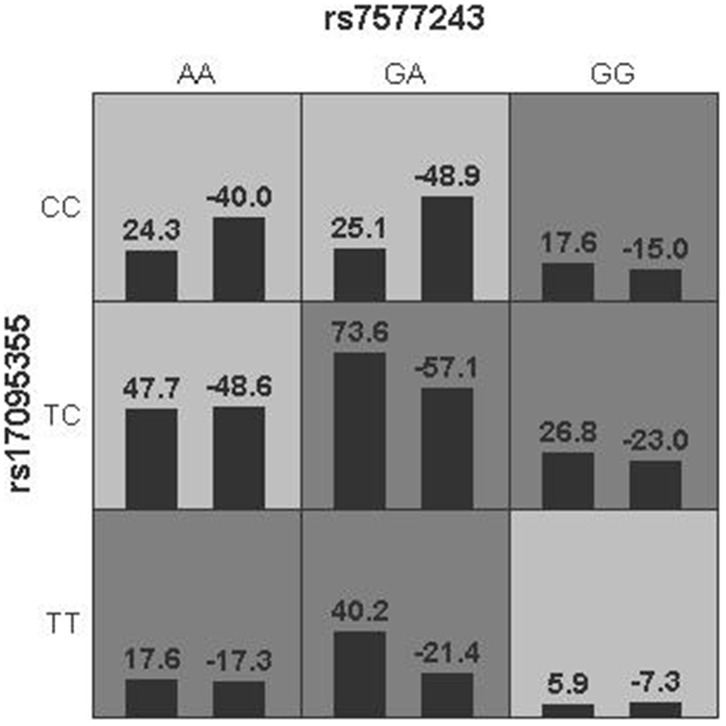
**Gene-gene interaction networks derived from GMDR regarding BA risk.** Multilocus genotype combinations of a two-factor model are associated with risk to BA best. In each cell, the left bar represents a positive score, and the right bar represents a negative score. High risk are represented by dark shading cells and low-risk cells by light shading. Rs17095355 was in *ADD3* region and rs7577243 was in *GPC1* region.

We further investigated the potential gene-gene interactions among SNPs in *ADD3*, *GPC1*, *ARF6* and *EFEMP1* using Generalized multifactor dimensionality reduction (GMDR) strategy (Figure 2 and Table 6). In terms of BA risk prediction, the best single factor model was *ADD3* (rs17095355) (*P* = 0.0012). The best two-factor model *ADD3* (rs17095355) - *GPC1* (rs7577243) was found significantly associated with BA (*P* = 0.0003). Besides, our result demonstrated that *ADD3* (rs17095355) - *GPC1* (rs7577243) - *EFEMP1* (rs11125609) was the best three-factor model and showed the most significant association (*P* < 0.0001; OR = 2.41, 95% CI = 1.68-3.46).

**Table 6:** Gene-gene interaction models contribution to BA risk by GMDR analysis.

**Number of factors**	**Best model ^a^**	**Training accuracy**	**Testing accuracy**	**CVC**	**Chi^2^**	***P* value**	**OR(95% CI)**
1	rs17095355	0.5663	0.5637	10/10	10.4962	0.0012	1.89(1.28-2.78)
2	rs7577243-rs17095355	0.5782	0.5216	6/10	12.8256	0.0003	1.94(1.35-2.79)
3	rs11125609-rs7577243-rs17095355	0.6072	0.5713	10/10	23.2364	<0.0001	2.41(1.68-3.46)

a. The best model was referred to as the one with the maximum testing accuracy and maximum cross-validation consistency (CVC). GMDR: generalized multifactor dimensionality reduction; OR: odds ratio; CI: confidence interval. Rs17095355, rs7577243 and rs11125609 were on *ADD3*, *GPC1* and *EFEMP1*, respectively.

**Figure 3:**
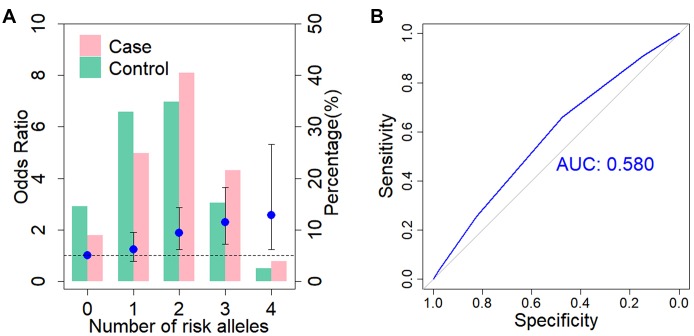
**Cumulative impact of two associated SNPs on BA risk.** (**A**) Distribution of cumulative risk alleles in BA cases (red) and controls (blue) for *ADD3* SNP rs17095355 and *GPC1* SNP rs6707262. The ORs are relative to group with zero risk alleles; vertical bars correspond to 95% confidence intervals. Horizontal line denotes the reference value (OR = 1.0). (**B**) Receiver operating characteristic (ROC) curve for assessment of the discriminative power of the risk prediction model. The area under curve (AUC) of the model is 0.58.

Lastly, we investigated whether there was a cumulative genetic effect with respect to the disease risk for *ADD3* SNP rs17095355 and *GPC1* SNP rs6707262 (Figure 3). The individuals can be divided into four classes according to the number of risk alleles that they carry (Figure 3A). There is an increase in ORs for BA occurrence with the increasing number of risk alleles against the baseline group of individuals carrying no risk alleles. Those carrying four risk alleles were more than twice as likely to have BA (OR = 2.56, 95% CI = 1.23-5.32; Supplementary Table 2) compared with those carrying none. We then evaluated the discriminatory power of a genetic test based on these two susceptibility SNPs by calculating the area under the receiver operating characteristic (ROC) curve, and the area under the curve (AUC) was estimated to be 0.58 (Figure 3B).

### Meta-analysis

**Figure 4:**
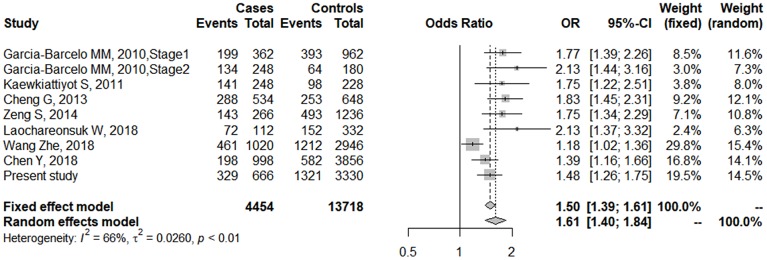
**Forest plot of meta-analysis for rs17095355 association with BA risk.** The sizes of the squares are proportional to study weights. Diamond markers indicated pooled effect sizes.

Literature searches and selection yielded 7 involved studies, which comprised 8 case-control studies [9, 11, 16–19, 21]. The study of Garcia-Barcelo MM, et al. included a GWAS stage and a replication stage in two independent samples [9], which were considered as two case-control studies in our meta-analysis (Table 7). Additionally, we included the data from the GWAS by Chen Y, et al [16] and the allele information of rs17095355 was obtained from the authors, which was imputed from the GWAS data with a info score of 0.998. The cases in the study of Tsai E.A et al [20] were part of samples from the study by Chen Y, et al, we therefore only included data from Chen Y, et al in the meta-analysis. Together with present study, a total of 9 case-control data consisting of 2,227 cases and 6859 controls was included in the meta-analysis (Figure 4). The risk allele T of has a higher frequency in Asians than in Europeans. The significant associations were consistent among 9 studies, although heterogeneity was found (*I^2^*=66%, *p* value <0.01, Figure 4). Therefore, the pooled OR was 1.61 (95% CI = 1.40-1.84) calculated by random effects model, which confirmed the association of rs17095355 with BA risk. In general, none of the studies produced a significantly biased result, but no obvious heterogeneity existed (*I^2^* = 3.5%, *p* value = 0.26) after the data sets of Laochareonsuk, W et al. (OR =2.13, 95% CI = 1.37-3.32) [19] and Wang Z, et al. (OR =1.18, 95% CI = 1.02-1.36) [21], were removed, which should be explained by the relatively larger and smaller OR values. The pooled OR of the remaining seven studies was 1.61 (95% CI = 1.48-1.76) calculated by fixed effects model.

**Table 7:** Summary of association studies for rs17095355 with BA susceptibility.

**Authors**	**Year**	**Ethnic group**	**Numbers**		**Frequencies of T allele**	
			**Cases**	**Controls**	**Cases**	**Controls**
Garcia-Barcelo MM, et al.	2010a	Chinese	181	481	0.551	0.409
Garcia-Barcelo MM, et al.	2010b	Chinese	124	90	0.539	0.355
Kaewkiattiyot S, et al.	2011	Thai	124	114	0.569	0.430
Cheng G, et al.	2013	Chinese	267	324	0.540	0.390
Tsai E.A, et al.	2014	Caucasian	171	1630	0.204	0.166
Zeng S, et al.	2014	Chinese	133	618	0.538	0.399
Laochareonsuk, W et al.	2018	Thai	56	166	0.643	0.458
Wang Z, et al.	2018	Chinese	510	1473	0.452	0.411
Chen Y, et al.	2018	Caucasian	499	1928	0.198	0.151
Present study	2019	Chinese	333	1665	0.494	0.397

### Functional annotation of associated SNPs

At *ADD3* locus, three associated SNPs (rs17095355, rs10509906 and rs2501577) were located in the intron region of *ADD3*. Rs17095355 and rs2501577 fall within a strong enhancer activity region (Supplementary Table 3) and they all alter the sequences of DNase I hypersensitivity sites and transcription factor binding motifs annotated by Roadmap (Supplementary Table 3). These three SNPs were expression quantitative trait loci (eQTLs) in multiple tissues from Genotype-Tissue Expression (GTEx) databases and were correlated with *ADD3* expression in immune system tissues including spleen and whole blood, where was thought to be involved in the progress of BA (Supplementary Figure 1). Of note, the risk allele T of rs17095355 was significantly associated the increased level of *ADD3* in spleen (*P* = 5.1×10^-13^, Supplementary Figure 1).

Rs6750380 and rs6707262 at 5’upstream of *GPC1* were located in a strong enhancer region as well as a site altering regulatory motifs and proteins bounding sites annotated by Roadmap (Supplementary Table 3). Rs6707262 was eQTL of *GPC1* in testis (*P* = 4.6 ×10^-11^) and tibial artery (*P* = 8.2×10^-6^; Supplementary Figure 2). Rs6750380 was also eQTL of *GPC1* in testis (*P* = 6.3×10^-15^) and cultured fibroblasts cells (*P* = 2.1×10^-4^; Supplementary Figure 3).

### Protein expression and epigenetic modification of associated genes

*In silico* analysis revealed that *ADD3* had a medium expression level in liver and a high expression level in gallbladder (Supplementary Figures 4A and 5). *GPC1* was not expressed in adult liver and gallbladder tissues (Supplementary Figures 4B and 5) *ADD3* showed significant difference in expression levels and methylation status between fetal and adult liver, with an approximately 2-fold higher expression level in fetal liver [25]. Four CpG sites located at *ADD3* gene region were differentially methylated when comparing the methylation patterns of the adult liver with the fetal liver [25].

### The protein-protein interaction (PPI) and co-expression results

**Figure 5:**
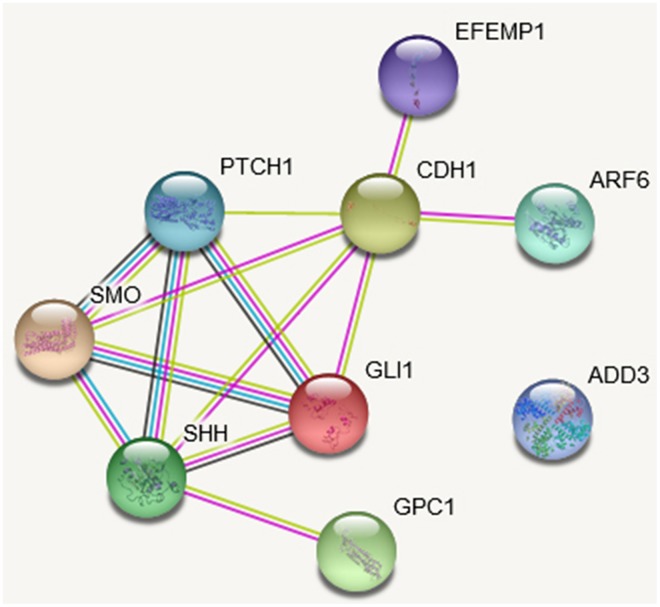
**The protein-protein interaction (PPI) network based on STRING database of studied genes.** The network is constructed for the four studies genes and Hedgehog pathway genes. The network nodes are proteins. The edges represent the predicted functional associations. An edge may be drawn with up to four different colored lines and these lines represent the existing associations that were predicted. A green line: neighborhood evidence; a blue line: cooccurrence evidence; a purple line: experimental evidence; a yellow line: textmining evidence; a black line: coexpression evidence.

Hedgehog signaling is an important mechanism in the pathology of BA and liver development. PPI analysis showed *GPC1*, *ARF6*, and *EFEMP1* gene interacted with Hedgehog pathway or related genes (Figure 5). *GPC1* was linked with Sonic Hedgehog (*SHH*) with experimentally determined evidence (Figure 5). Experimentally determined evidence also demonstrated that *ARF6* and *EFEMP1* gene were interacted with cadherin 1 (*CDH1*), which was linked to Hedgehog pathway members glioma-associated oncogene homolog 1 (*GLI1*), *SHH* and smoothened (*SMO*) (Figure 5). Although knockdown of *add3* activated the Hedgehog pathway in zebrafish larvae, no recognized link between *ADD3* and the Hedgehog pathway was found.

## DISCUSSION

We performed association analysis for four BA susceptibility genes of discovered in recent GWASs. Our results validated that three *ADD3* variants (rs17095355, rs10509906 and rs2501577), and two *GPC1* variants (rs6750380 and rs6707262) were associated with BA susceptibility in Chinese population. Meta-analysis for rs17095355 association with BA further confirmed the association in Asian and Caucasian population. Associations of *ARF6* and *EFEMP1* SNPs were not replicated in current sample-set.

The 10q24.2 region encompassing *ADD3* and *XPNPEP1* genes was found association in a GWAS of Chinese population, and further fine-mapping of this region identified *ADD3* as the susceptibility gene [9, 17]. Morpholino antisense oligonucleotide (MO) knockdown targeting *add3a* in zebrafish, not *xpnpep1*, produced intrahepatic defects and decreased biliary function [22]. The risk allele T of the top SNP rs17095355 was found association with decreased level of *ADD3* in BA liver tissues, but no such correlation was found for *XPNPEP1* [17]. Rs17095355 was also found to act as an eQTL for *ADD3* in whole blood and spleen from the GTEx database. These foundings indicated that *ADD3* was the BA susceptibility gene at 10q24.2. The association between rs17095355 of *ADD3* and BA was investigated repeatedly in multiple studies from different population [11, 18–21], and a meta-analysis comprising six case-control studies before 2015 has been conducted [26]. We incorporated the published data before 2015, the newly published data and our current data to perform a further meta-analysis. In Asian population, rs17095355 showed consistent significant association with BA [11, 18, 19, 21]. Rs17095355 also showed significant association in European descent, but rs7099604 showed more significant association [20]. These evidences revealed *ADD3* as a common susceptibility gene in Asian and Caucasian population. The risk allele T of rs17095355 was more frequent in Asian than in Europe decedents, which might contribute to the higher incidence of BA in Asian.

*ADD3* encodes adducin-γ belonging to Adducin family. Adducins are heteromeric membrane skeletal proteins composed of different subunits referred to as adducin alpha, beta and gamma. Adducin-γ are ubiquitously expressed and abundantly expressed in biliary epithelia [17]. Adducins are involved in the assembly of spectrin-actin network in erythrocytes and at sites of cell-cell contact in epithelial tissues. Notably, the functional roles of adducins in remodeling of epithelial junctions during embryonic morphogenesis indicated that adducins might be involved in the biliary pathology in BA [27]. Morpholino-mediated knockdown of *add3* activated the Hedgehog pathway in zebrafish larvae, providing a previously unrecognized link between *ADD3* and the Hedgehog pathway [17]. It has long been recognized that BA is characterized by excessive Hedgehog pathway activity, which stimulated biliary epithelial-mesenchymal transitions (EMT) and might contribute to biliary dysmorphogenesis during liver development [28]. The underlying molecular mechanisms though which *ADD3* regulates Hedgehog signaling needs further exploration.

Rare copy number variants and common variants of *GPC1* both contributed to BA risk [10, 23, 24]. We genotyped ten tag SNPs in the current sample-set and confirmed *GPC1* association with BA risk. Two new associated SNPs were identified (rs6750380 and rs6707262), which also had eQTL effects on *GPC1*. *GPC1* encodes glypican-1, one of six members of the glypican family, which attach to the cell membrane by a glycosyl-phosphatidylinositol linkage. Previous study showed that glypican-1 was located in the apical membrane of cholangiocytes and had reduced levels in diseased liver from BA patients [23]. Knockdown of *gpc1* in zebrafish led to developmental biliary defects resembling BA and Hedgehog activity was increased in the livers *of gpc1* morphants [23]. Glypican-3 (*GPC3*) acted as a negative regulator of Hedgehog signaling, through interacting with high affinity with Hedgehog and competing with Patched for Hedgehog binding [29]. Together, these findings suggest *GPC1* could act as an inhibitor for Hedgehog ligands via the similar mechanisms as *GPC3*.

A GWAS in Caucasian identified *ARF6* as a susceptibility gene at 14q21.3 [12]. *ARF6* shows a medium expression level in liver and gallbladder (Supplementary Figures 4C and 5). Knockdown of the two zebrafish homologs resembled the syndromes of BA, which indicated that *arf6* was required in early biliary development [12]. The frequency of rs3126184 risk allele in Caucasian controls was 0.13, but only 0.037 in current controls. The association was not validated in our samples. Since only two reported SNPs were studied, we could not preclude the possibility that other variants of *ARF6* were associated with BA risk. Another explanation for lack replication of the association might be the genetic heterogeneity, that *ARF6* might be a Caucasian specific susceptibility gene.

*EFEMP1* mapping to chromosome 2p16, encodes epidermal growth factor-containing fibulin-like extracellular matrix protein 1, which is also known as Fibulin-3. Its main role is to maintain basement membrane stability and extracellular matrix integrity, which is implicated in cell proliferation and organogenesis [16, 30, 31]. *EFEMP1* is also a major extracellular matrix protein involving in the biological process of fibrosis [32]. The expression level of *EFEMP1* was higher in BA patients than in controls [16]. Together, these findings suggest a potential role for *EFEMP1* in the pathogenesis of BA. A cluster of SNPs within *EFEMP1* gene were significantly associated with BA susceptibility in a recent GWAS in Europeans [16]. Four tag SNPs in the current study did not reach the significance level, however, showed the same effect direction as in the original study [16]. Given the moderate effects of this locus, our sample was not large enough to detect the association. Therefore, further studies were needed to validate this association in other independent samples.

In summary, we confirmed association of variants in *ADD3* and *GPC1* with BA susceptibility in Chinese population. The interaction of SNPs in disease-associated genes contributed to BA susceptibility. Bioinformatics analysis revealed that the risk SNPs influenced the expression of susceptibility genes.

## MATERIALS AND METHODS

### Subjects

A total of 340 unrelated patients were recruited. Diagnose of BA was based on clinical manifestations, laboratory tests, imaging examinations and ultimately confirmed by cholangiography. Patients with other associated congenital malformations were excluded from the study. Clinical information of patients was shown in Table 1. Totally, 1,665 unrelated healthy individuals without BA, other congenital diseases, autoimmune, or liver disease were enrolled as controls. All participants were biologically unrelated Chinese Han individuals and were recruited at Xinhua hospital affiliated to Shanghai Jiao Tong University School of Medicine from 2008 to 2018. Peripheral blood samples were collected in a standard EDTA tube for DNA extraction and all data was recorded anonymously. Genomic DNA was extracted from peripheral blood leukocytes using QIAamp DNA Blood Mini Kit according to the manufacturer's protocol (Qiagen, Hilden, Germany). Written informed consent was obtained from all participants or their parents. This study was conducted in accordance with the Declaration of Helsinki (version 2002) and was approved by the institution review board of Xinhua Hospital affiliated to Shanghai Jiao Tong University School of Medicine.

### SNP selection

A GWAS in Chinese population revealed BA association with 10q24.2 region encompassing *ADD3* and *XPNPEP1* [9]. Subsequent fine-mapping indicated that a risk haplotype, consisting of five SNPs: rs17095355, rs10509906, rs2501577, rs6584970, and rs7086057, could capture the 10q24.2 risk alleles [17]. Among the five SNPs, rs2501577, rs6584970 and rs7086057 were in high LD (r^2^ ≥ 0.98). Therefore, we select rs17095355, rs10509906 and rs2501577 for replication analysis. We selected 10 tag SNPs from South Han Chinese data in 1000 genomes project database to cover the common variation in *GPC1* gene region. Rs2292832 failed in the assay. Two SNPs (rs3126184 and rs10140366) in perfect LD 3’ upstream of *ARF6* were reported association with BA in Caucasian children [12]. We genotyped these two SNPs in our samples, but rs10140366 failed in the assay. About 13 SNPs in high LD within *EFEMP1* region on 2p16.1 were associated with BA susceptibility in a European-American cohort [16]. We selected 4 tag SNPs including the top SNP (rs10865291) for replication.

### SNP genotyping

Genotyping was performed using the Fluidigm 96.96 Dynamic Array IFCs (Fluidigm, San Francisco, CA, United States) [33]. Cases and controls were plated out in sets of 96 samples and combined into 384-well arrays for genotyping. Polymerase chain reaction (PCR) was performed in a 5-μl reaction and cycling conditions were set using the standard procedure according to the manufacturer's protocol. To obtain genotype calls, we analyzed the data using EP1 SNP Genotyping Analysis software. The software defined the genotype of each sample based on the relative fluorescence intensities.

### Functional annotation

We first investigated the functional consequences of the associated SNP by checking HaploRegv4.1 database. To examine whether the associated SNPs were eQTL, we made inquiries in GTEx Analysis Release V8 (dbGap Accession phs000424.v8.p2) [34]. The GTEx project collected and analyzed multiple human tissues from donors who were densely genotyped to assess genetic variation within their genomes. By analyzing global RNA expression within individual tissues and treating the expression levels of genes as quantitative traits, variations in genes expression that are highly correlated with genetic variation can be identified as eQTL.

### Meta-analysis

Since 2010 when 10q24.2 region was implicated association with BA in, rs17095355 was repeatedly genotyped in the following studies, thus we performed a meta-analysis of rs17095355 association with BA risk. In order to find eligible studies, we searched PubMed using combinations of the following terms: “*ADD3*” or “adducin 3” or “*XPNPEP1*” or “X-prolyl aminopeptidase 1” and “biliary atresia” and “association”. We also searched the reference list of review articles and lists of publications of researchers working in this field. The included data covered all English-language publications up to October 2019. Meta-analysis was conducted using the Meta package in R (http://cran.r-project.org/web/packages/meta/index.html) [35]. The *I*^2^ was calculated to quantify the magnitude of between-study heterogeneity and the Cochrane Q statistic was used to determine significance for heterogeneity. An *I*^2^ of 25%, 50%, and 75% represents low, medium, and large heterogeneity, respectively.

### *In silico* protein expression and epigenetic analysis

We searched for the expression pattern of studied genes in THE HUMAN PROTEIN ATLAS (https://www.proteinatlas.org/). The immunohistochemistry results in liver and gallbladder tissues were extracted. EWAS Catalog β (http://www.ewascatalog.org/) was used as a lookup for epigenetic modificaton of studied genes.

### PPI network construction

We explored PPI using STRING database (http://string-db.org/) [36]. Four studied genes (*ADD3*, *GPC1*, *ARF6*, and *EFEMP1*) and Hedgehog pathway genes were used to query STRING database. The PPI relationships were analyzed on the STRING database with the required confidence (combined score) > 0.4 as the threshold. After the PPIs were searched, the PPI network was constructed on STRING website.

### Statistical analysis

Quality control was performed using PLINK 1.09 [37]. HWE of each SNP in both case and control groups was tested. Four genetic models, including the allelic, additive, dominant and recessive model, together with a genotypic association test (2df test) were used to analyze the association for each SNP using PLINK 1.09 [37]. We calculated per allele OR and 95%CI. We calculated LD between SNPs and constructed haplotype block using Haploview4.2 [38]. Haplotype phasing was performed using SHAPEIT and haplotype association was tested using R package [39]. Conditional logistic analysis was performed to find additional markers with independent effect by adding the top associated markers as covariates in logistic regression. The study-wide significance threshold for SNP association analysis is *P* = 0.027 (0.05/18). Gene-gene interactions were investigated using GMDR software Beta 0.9 [40]. *ADD3* SNP rs17095355 and *GPC1* SNP rs6707262 were used to build the risk assessment model. The genotypes of each SNP were coded as 0, 1, or 2 indicating the number of risk alleles in one individual. The cumulative genetic risk score of each individual is the sum of rsik alleles from the two SNPs (score range, 0 - 4). To test the prediction capability of the model, we generated the ROC curve and calculated the AUC using the pROC R package [41].

## Supplementary Material

Supplementary Figures

Supplementary Tables
