# Supplementary material for: Association of common variation in ADD3 and GPC1 with biliary atresia susceptibility
Source: Aging (Albany NY). 2020 Apr 21;12(8):7163–82. doi: 10.18632/aging.103067 (PMC7202506; doi:10.18632/aging.103067)
Supplement: Supplementary Figures [file aging-12-103067-s002..pdf]

SUPPLEMENTARY FIGURES

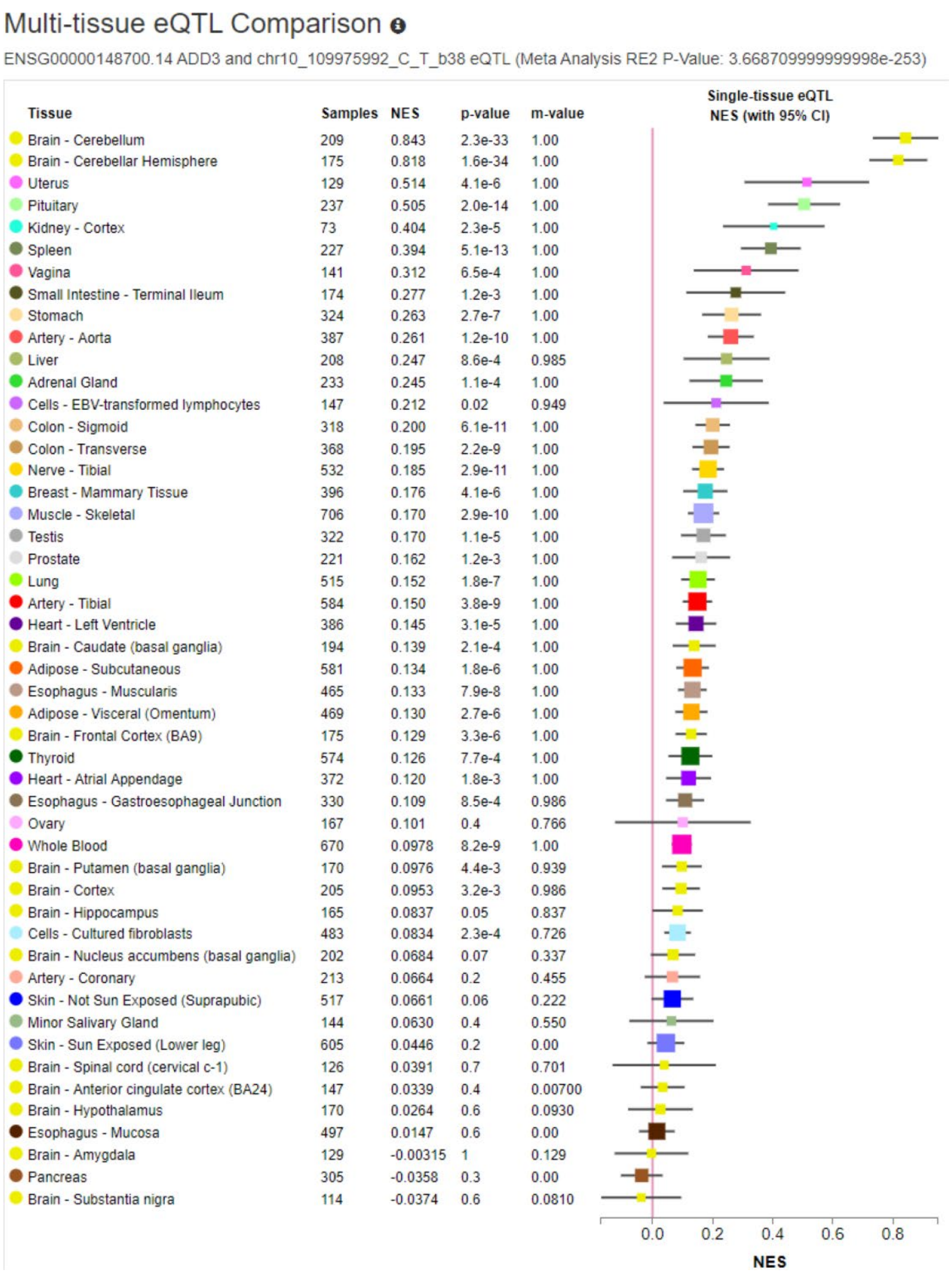

Supplementary Figure 1. Multi-tissue eQTL plot for rs17095355 on *ADD3* expression. The plot was based on genotype and transcriptome data from the GTEx Analysis Release V8 (dbGaP Accession phs000424.v8.p2). The modeled allele is rs17095355 risk allele T.

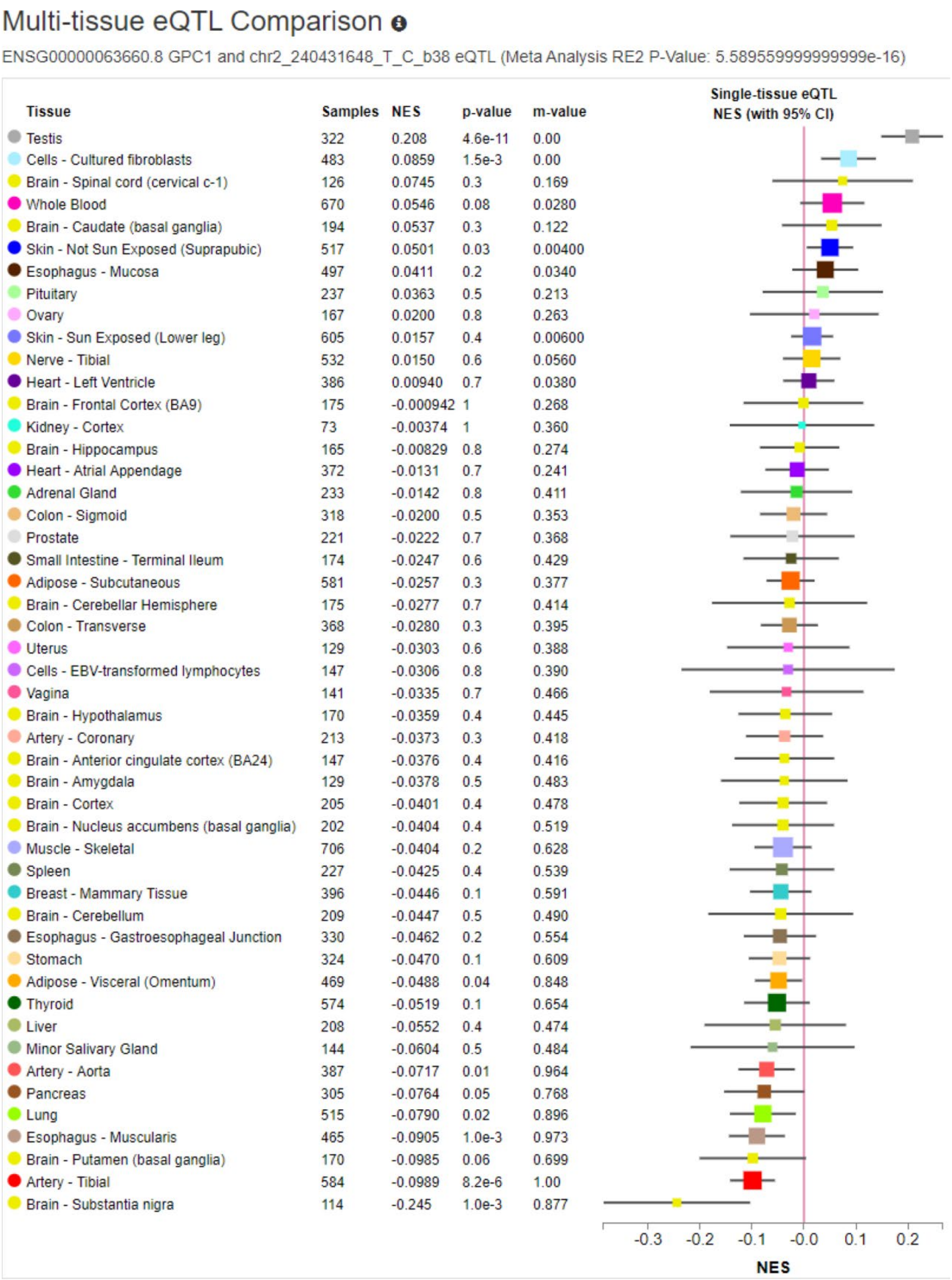

Tissue	Samples	NES	p-value	m-value
Testis	322	0.208	4.6e-11	0.00
Cells - Cultured fibroblasts	483	0.0859	1.5e-3	0.00
Brain - Spinal cord (cervical c-1)	126	0.0745	0.3	0.169
Whole Blood	670	0.0546	0.08	0.0280
Brain - Caudate (basal ganglia)	194	0.0537	0.3	0.122
Skin - Not Sun Exposed (Suprapubic)	517	0.0501	0.03	0.00400
Esophagus - Mucosa	497	0.0411	0.2	0.0340
Pituitary	237	0.0363	0.5	0.213
Ovary	167	0.0200	0.8	0.263
Skin - Sun Exposed (Lower leg)	605	0.0157	0.4	0.00600
Nerve - Tibial	532	0.0150	0.6	0.0560
Heart - Left Ventricle	386	0.00940	0.7	0.0380
Brain - Frontal Cortex (BA9)	175	-0.000942	1	0.268
Kidney - Cortex	73	-0.00374	1	0.360
Brain - Hippocampus	165	-0.00829	0.8	0.274
Heart - Atrial Appendage	372	-0.0131	0.7	0.241
Adrenal Gland	233	-0.0142	0.8	0.411
Colon - Sigmoid	318	-0.0200	0.5	0.353
Prostate	221	-0.0222	0.7	0.368
Small Intestine - Terminal Ileum	174	-0.0247	0.6	0.429
Adipose - Subcutaneous	581	-0.0257	0.3	0.377
Brain - Cerebellar Hemisphere	175	-0.0277	0.7	0.414
Colon - Transverse	368	-0.0280	0.3	0.395
Uterus	129	-0.0303	0.6	0.388
Cells - EBV-transformed lymphocytes	147	-0.0306	0.8	0.390
Vagina	141	-0.0335	0.7	0.466
Brain - Hypothalamus	170	-0.0359	0.4	0.445
Artery - Coronary	213	-0.0373	0.3	0.418
Brain - Anterior cingulate cortex (BA24)	147	-0.0376	0.4	0.416
Brain - Amygdala	129	-0.0378	0.5	0.483
Brain - Cortex	205	-0.0401	0.4	0.478
Brain - Nucleus accumbens (basal ganglia)	202	-0.0404	0.4	0.519
Muscle - Skeletal	706	-0.0404	0.2	0.628
Spleen	227	-0.0425	0.4	0.539
Breast - Mammary Tissue	396	-0.0446	0.1	0.591
Brain - Cerebellum	209	-0.0447	0.5	0.490
Esophagus - Gastroesophageal Junction	330	-0.0462	0.2	0.554
Stomach	324	-0.0470	0.1	0.609
Adipose - Visceral (Omentum)	469	-0.0488	0.04	0.848
Thyroid	574	-0.0519	0.1	0.654
Liver	208	-0.0552	0.4	0.474
Minor Salivary Gland	144	-0.0604	0.5	0.484
Artery - Aorta	387	-0.0717	0.01	0.964
Pancreas	305	-0.0764	0.05	0.768
Lung	515	-0.0790	0.02	0.896
Esophagus - Muscularis	465	-0.0905	1.0e-3	0.973
Brain - Putamen (basal ganglia)	170	-0.0985	0.06	0.699
Artery - Tibial	584	-0.0989	8.2e-6	1.00
Brain - Substantia nigra	114	-0.245	1.0e-3	0.877

Supplementary Figure 2. Multi-tissue eQTL plot for rs6707262 on *GPC1* expression. The plot was based on genotype and transcriptome data from the GTEx Analysis Release V8 (dbGaP Accession phs000424.v8.p2). The modeled allele is rs6707262 risk allele G.

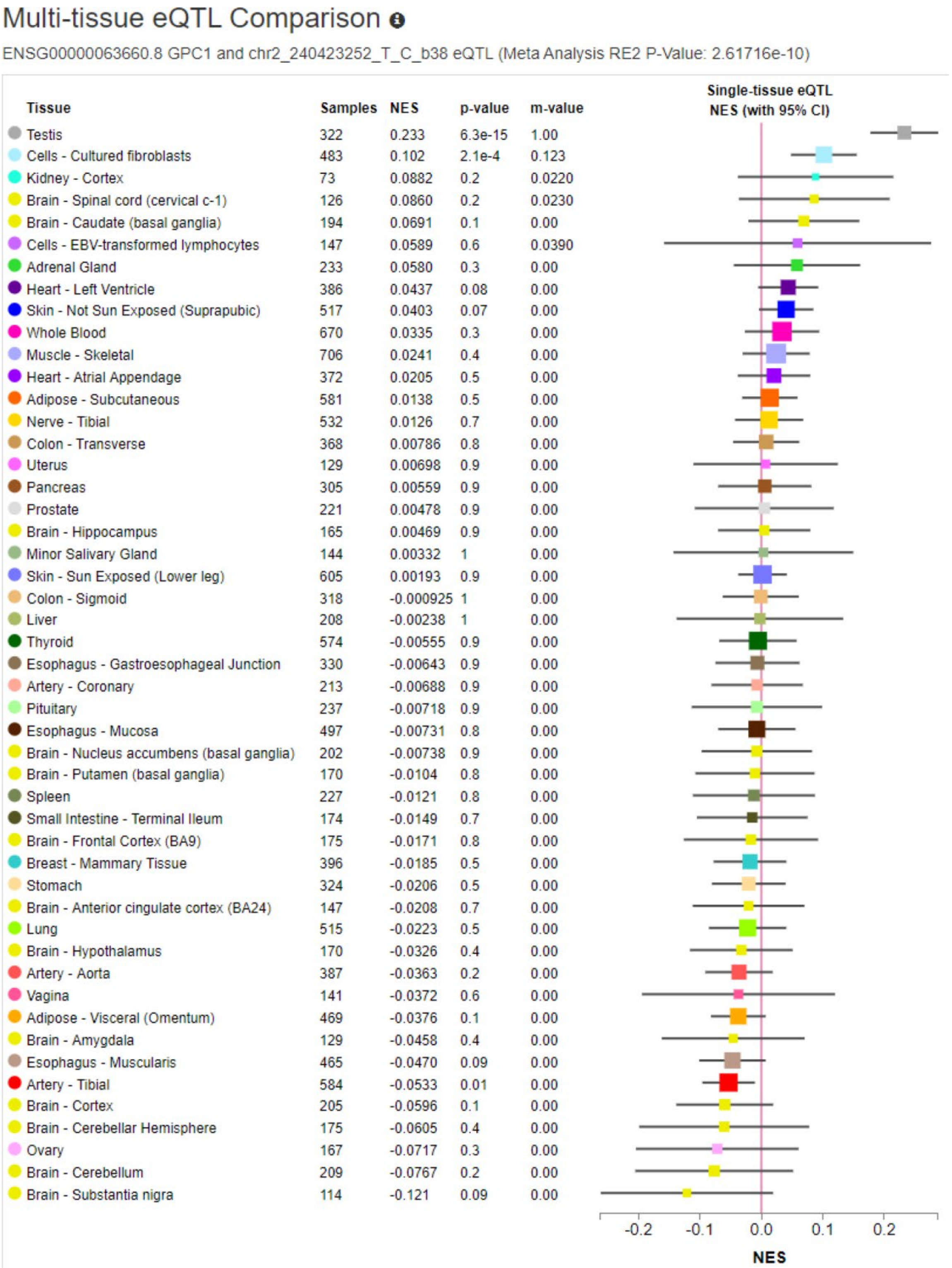

Tissue	Samples	NES	p-value	m-value
Testis	322	0.233	6.3e-15	1.00
Cells - Cultured fibroblasts	483	0.102	2.1e-4	0.123
Kidney - Cortex	73	0.0882	0.2	0.0220
Brain - Spinal cord (cervical c-1)	126	0.0860	0.2	0.0230
Brain - Caudate (basal ganglia)	194	0.0691	0.1	0.00
Cells - EBV-transformed lymphocytes	147	0.0589	0.6	0.0390
Adrenal Gland	233	0.0580	0.3	0.00
Heart - Left Ventricle	386	0.0437	0.08	0.00
Skin - Not Sun Exposed (Suprapubic)	517	0.0403	0.07	0.00
Whole Blood	670	0.0335	0.3	0.00
Muscle - Skeletal	706	0.0241	0.4	0.00
Heart - Atrial Appendage	372	0.0205	0.5	0.00
Adipose - Subcutaneous	581	0.0138	0.5	0.00
Nerve - Tibial	532	0.0126	0.7	0.00
Colon - Transverse	368	0.00786	0.8	0.00
Uterus	129	0.00698	0.9	0.00
Pancreas	305	0.00559	0.9	0.00
Prostate	221	0.00478	0.9	0.00
Brain - Hippocampus	165	0.00469	0.9	0.00
Minor Salivary Gland	144	0.00332	1	0.00
Skin - Sun Exposed (Lower leg)	605	0.00193	0.9	0.00
Colon - Sigmoid	318	-0.000925	1	0.00
Liver	208	-0.00238	1	0.00
Thyroid	574	-0.00555	0.9	0.00
Esophagus - Gastroesophageal Junction	330	-0.00643	0.9	0.00
Artery - Coronary	213	-0.00688	0.9	0.00
Pituitary	237	-0.00718	0.9	0.00
Esophagus - Mucosa	497	-0.00731	0.8	0.00
Brain - Nucleus accumbens (basal ganglia)	202	-0.00738	0.9	0.00
Brain - Putamen (basal ganglia)	170	-0.0104	0.8	0.00
Spleen	227	-0.0121	0.8	0.00
Small Intestine - Terminal Ileum	174	-0.0149	0.7	0.00
Brain - Frontal Cortex (BA9)	175	-0.0171	0.8	0.00
Breast - Mammary Tissue	396	-0.0185	0.5	0.00
Stomach	324	-0.0206	0.5	0.00
Brain - Anterior cingulate cortex (BA24)	147	-0.0208	0.7	0.00
Lung	515	-0.0223	0.5	0.00
Brain - Hypothalamus	170	-0.0326	0.4	0.00
Artery - Aorta	387	-0.0363	0.2	0.00
Vagina	141	-0.0372	0.6	0.00
Adipose - Visceral (Omentum)	469	-0.0376	0.1	0.00
Brain - Amygdala	129	-0.0458	0.4	0.00
Esophagus - Muscularis	465	-0.0470	0.09	0.00
Artery - Tibial	584	-0.0533	0.01	0.00
Brain - Cortex	205	-0.0596	0.1	0.00
Brain - Cerebellar Hemisphere	175	-0.0605	0.4	0.00
Ovary	167	-0.0717	0.3	0.00
Brain - Cerebellum	209	-0.0767	0.2	0.00
Brain - Substantia nigra	114	-0.121	0.09	0.00

Supplementary Figure 3. Multi-tissue eQTL plot for rs6750380 on *GPC1* expression. The plot was based on genotype and transcriptome data from the GTEx Analysis Release V8 (dbGaP Accession phs000424.v8.p2). The modeled allele is rs6750380 risk allele G.

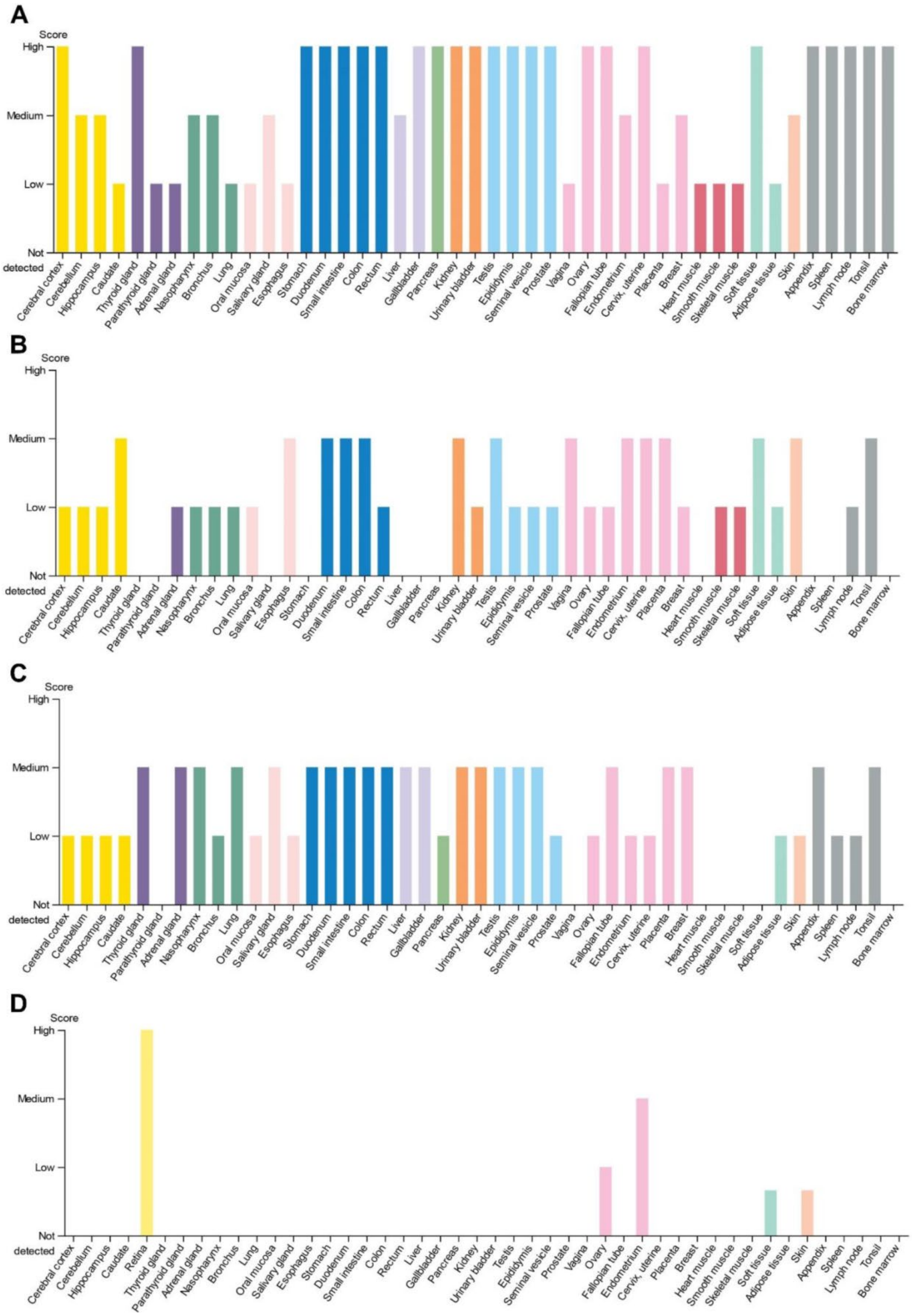

Supplementary Figure 4. The expression of *ADD3*, *GPC1*, *ARF6* and *EFEMP1* in human tissues. (**A**–**D**) stand for *ADD3*, *GPC1*, *ARF6* and *EFEMP1* expression respectively in human tissues from THE HUMAN PROTEIN ATLAS (https://www.proteinatlas.org/). The vertical axis indicates the expression level of the gene in each tissue.

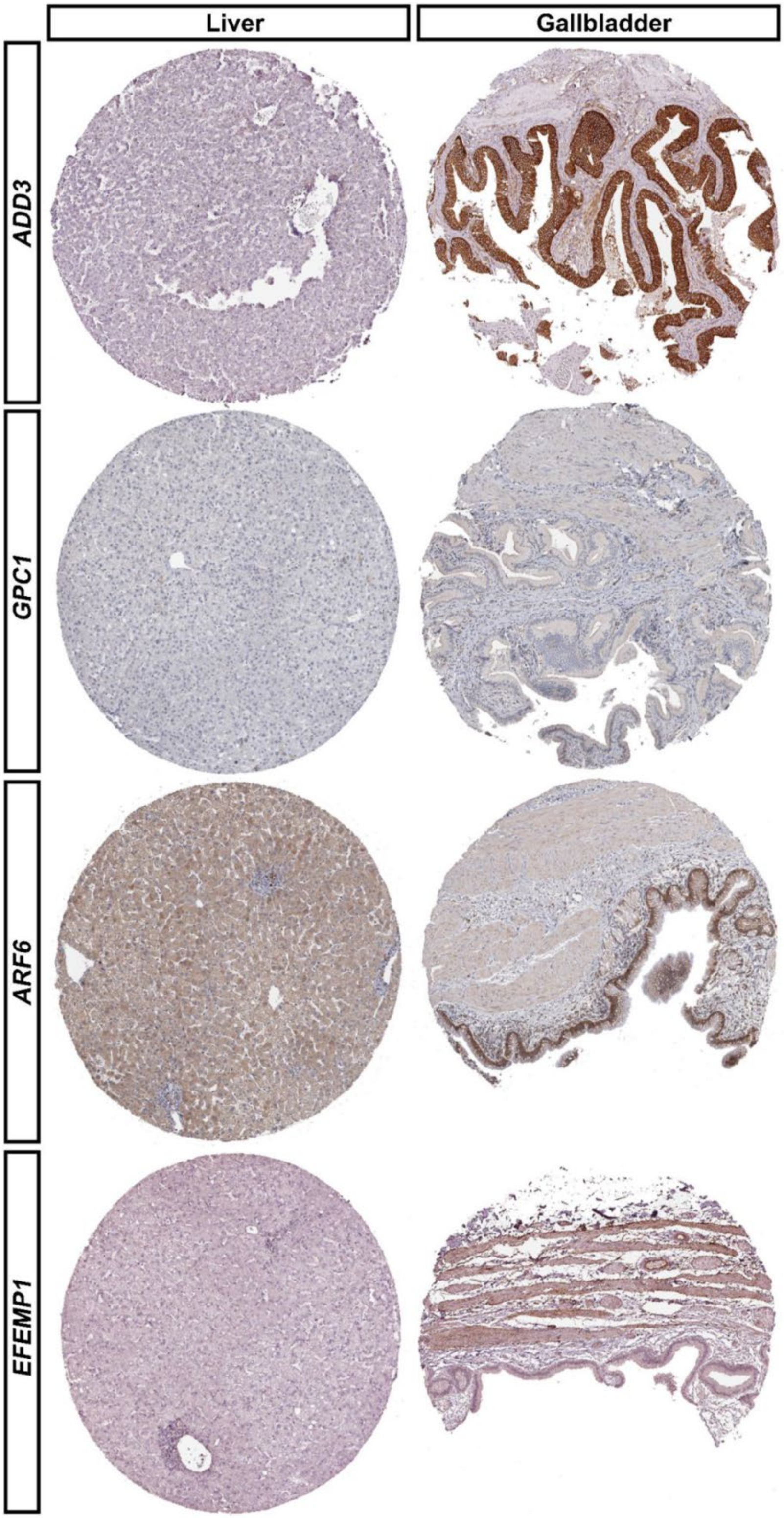

Supplementary Figure 5. Immunohistochemistry analysis of *ADD3*, *GPC1*, *ARF6* and *EFEMP1* in adult human liver and gallbladder tissues from THE HUMAN PROTEIN ATLAS database (https://www.proteinatlas.org/).
