# Supplementary material for: Association of common variation in ADD3 and GPC1 with biliary atresia susceptibility
Source: Aging (Albany NY). 2020 Apr 21;12(8):7163–82. doi: 10.18632/aging.103067 (PMC7202506; doi:10.18632/aging.103067)
Supplement: Supplementary Tables [file aging-12-103067-s001..pdf]

SUPPLEMENTARY TABLES

Supplementary Table 1. Allele frequencies of four Tag SNPs of *EFEMP1* in Europeans and Chinese.

	Alleles	Risk allele	Risk allele frequency		Ethnic group	Study
			cases	controls		
rs1346786	T/C	T	0.39	0.29	Europeans	Chen Y, 2018, stage1
			0.35	0.30	Europeans	Chen Y, 2018, stage2
			0.88	0.86	Chinese	Present study
rs11125609	C/T	C	0.36	0.28	Europeans	Chen Y, 2018, stage1
			0.31	0.29	Europeans	Chen Y, 2018, stage2
			0.56	0.54	Chinese	Present study
rs10865291	A/G	A	0.43	0.33	Europeans	Chen Y, 2018, stage1
			0.40	0.32	Europeans	Chen Y, 2018, stage2
			0.80	0.78	Chinese	Present study
rs1430193	T/A	T	0.44	0.34	Europeans	Chen Y, 2018, stage1
			0.42	0.33	Europeans	Chen Y, 2018, stage2
			0.09	0.09	Chinese	Present study

Supplementary Table 2. Number of accounted risk alleles in BA cases and controls and ORs for BA by cumulative risk alleles.

Number of risk alleles	Control	Case	OR(95%CI)	*P* value
0	14.6%	9.0%	1	
1	32.9%	24.9%	1.23(0.79,1.91)	3.71E-01
2	34.9%	40.5%	1.88(1.23,2.87)	3.61E-03
3	15.2%	21.6%	2.30(1.45,3.65)	4.01E-04
4	2.5%	3.9%	2.56(1.23,5.32)	1.20E-02

OR: odds ratio; CI: confidence interval.

Supplementary Table 3. Functional annotation of SNPs correlated with newly identified risk variants using data from HaploReg v4.1.

SNP	Position	Promoter histone marks[b]	Enhancer histone marks[c]	DNAse[d]	Proteins bound[e]	Motifs changed[f]
rs6750380	241362669		ESDR, CRVX		JUND, FOSL2	
rs6707262	241371065		4 tissues			5 altered motifs
rs17095355	111735750		11 tissues	5 tissues		Hoxa5, XBP-1
rs10509906	111757674			ESDR		Ets,Gfi1, Gfi1b
rs2501577	111846687	BLD	7 tissues	IPSC, BLD, BLD		BDP1, TBX5

SNP: Single Nucleotide Polymorphism; The chromosome position (bp) is based on GRCH37;
ESDR: H9 Derived Neuronal Progenitor Cultured Cells or H9 Derived Neuron Cultured Cells; CRVX: HeLa-S3 Cervical Carcinoma Cell Line; IPSC: iPS DF 6.9 Cells; BLD: Primary T cells from peripheral blood or Primary Natural Killer cells from peripheral blood.
b. Evidence of local H3K4Me1 and H3K27Ac modification (cell lines/types: if >3, only the number is included).
c. Evidence of local H3K4Me3 modification (cell lines/types: if >3, only the number is included).
d. Evidence of chromatin hypersensitivity to DNase (cell lines/types: if >3, only the number is included).
e. ChIP-seq experiments indicate alteration in binding of transcription factor (if >3, only the number is included).
f. Evidence of alteration in regulatory motif (if >3, only the number is included).
